# Mapping of national population-based surveys for better reporting of health-related indicators in the Eastern Mediterranean Region

**DOI:** 10.1186/s12889-023-15330-6

**Published:** 2023-03-26

**Authors:** Sahand Riazi-Isfahani, Henry Victor Doctor, Eman Abdelkreem Aly, Hanem Mohamed Basha, Reza Majdzadeh, Arash Rashidian

**Affiliations:** 1 National Institute of Health Research (NIHR), Tehran University of Medical Sciences, Tehran, Iran; 2 World Health Organization, Division of Science, Information and Dissemination, World Health Organization, Regional Office for the Eastern Mediterranean, Cairo, Egypt; 3 Department of Health Emergencies, World Health Organization, Regional Office for the Eastern Mediterranean, Cairo, Egypt; 4 School of Health and Social Care, University of Essex, Colchester, England

**Keywords:** Eastern mediterranean region, Population-based surveys, Sustainable development goals

## Abstract

**Background:**

Population-based surveys are the main data source to generate health-related indicators required to monitor progress toward national, regional and global goals effectively. Although the Eastern Mediterranean Region of World Health Organization (WHO) member states conduct many population-based surveys, they are not led regularly and fail to provide relevant indicators appropriately. Therefore, this study aims two-fold: to map out population-based surveys to be conducted data for the health-related indicators in the Region and propose a timetable for conducting national population-based surveys in the Region.

**Methods:**

The study was conducted in six phases: 1) Selecting survey-based indicators; 2) Extracting and comparing relevant survey modules; 3) Identifying sources of data for the indicators; 4) Assessing countries' status in reporting on core health indicators; 5) Review and confirmation of the results by the experts.

**Results:**

Population-based surveys are the sources of data for 44 (65%) out of 68 regional core health indicators and two (18%) out of 11 health-related Sustainable Development Goals (SDG) 3 indicators. The Health Examination Survey (HES) could cover 65% of the survey-based indicators. A total of 91% of survey-based indicators are obtained by a combination of HES, Demographic and Health Survey (DHS), Multiple Indicator Cluster Survey (MICS) and Global School-based Student Health Survey (GSHS).

**Conclusion:**

In order to effectively report health-related indicators, HES, DHS/MICS and GSHS are considered essential in national survey timetables. Each country needs to devise and implement a plan for population-based surveys by considering factors such as national health priorities, financial and human capacities, and previous experiences.

## Background

Reliable and timely information is essential for monitoring progress toward national, regional and international health-related goals and developing and evaluating health-related policies, including identifying national health priorities, needs and effective resource allocation [1–6]. In order to support the Member States in effectively monitoring the health situation, the WHO Regional Office for Eastern Mediterranean worked with the Member States of the Region since 2012 to develop a framework for health information systems (HIS) and 68 health core indicators [7]. These core indicators focus on three components: health determinants and risks, health status, morbidity and cause-specific mortality, and health system response. The HIS framework was endorsed during the 61st session of the WHO Regional Committee for the Eastern Mediterranean in 2014. Since then, WHO reports annually on the core indicators and verifies data with the Member States. The HIS framework also covers indicators for monitoring the progress toward Universal Health Coverage (UHC) and health-related Sustainable Development Goals (SDG) [5, 8]. Data to generate the regional core health indicators come from two main sources: registration systems (i.e. surveillance and administrative data) and institution-based or population-based surveys [9].

The Eastern Mediterranean Region (EMR) is a heterogeneous region not only in geopolitical and social context, ethnicity and languages spoken but also in socioeconomic and health profiles. For example there is more than 24 years difference in life expectancies between Somalia (56.5 years) and Kuwait (81.0 years) [10]. Financial resources allocated to the health systems also vary broadly across countries, with the lowest and highest recorded values for per capita current health expenditure (CHE) of 50 USD in Afghanistan and 1817 USD in the United Arab Emirates in 2018 [10].

Moreover, conflicts and terrorism have caused massive humanitarian crises in the Region and disrupted health systems' structures and functions, mostly affecting Afghanistan, Iraq, Libya, Palestine, Somalia, Syria, and Yemen [11, 12].

As a result, there are huge differences between health systems' performances and capacities among the countries. While some countries have well-established health systems and can mobilize national financial and technical resources to strengthen their HIS, others rely only on international funds and technical support [13, 14]. Countries of the Region can be categorized into three groups according to World Bank country classifications by income level [15]. Group 1, or high-income countries, consists of countries where socioeconomic development has progressed considerably over the past decades. These countries are Bahrain, Kuwait, Oman, Qatar, Saudi Arabia, and the United Arab Emirates. Group 2, or upper-middle and lower-middle income countries, consists of Egypt, the Islamic Republic of Iran, Iraq, Jordan, Lebanon, Libya, Morocco, occupied Palestinian territory, the Syrian Arab Republic, and Tunisia. Although these countries have developed infrastructure for HIS in recent years, they might face resource constraints. Group 3 or low-income countries, including Afghanistan, Djibouti, Pakistan, Somalia, Sudan and Yemen, face major constraints in improving their health information systems due to limited resources, political instability, and other complex development challenges.

Many national population-based and institution-based surveys have already conducted by the Region’s countries (Table 1) but the health-related indicators that can be obtained from the surveys were not reported. An assessment conducted by Alwan et al. in 2016 of the countries' capacity to report on core indicators in the Region showed that due to lack of a national comprehensive plan, the population-based surveys failed to appropriately provide relevant indicators [9].

**Table 1:** National population-based and institution-based surveys conducted by the Eastern Mediterranean Region countries and the year of conducting the last survey

	**Country**	**Demographic and Health Survey (DHS)**	**Multiple Indicator Cluster Survey (MICS)**	**Non-communicable Disease Risk Factors Survey (STEPS)**	**Household Expenditure Survey**	**Service Availability and Readiness Assessment (SARA)**	**Global Adult Tobacco Survey (GATS)**	**Global school-based student health survey (GSHS)**
1	Afghanistan	2015	2010–11		^a^			2014
2	Bahrain			2007	2006			2016
3	Djibouti		2006		2013	2015		2006
4	Egypt	2015	2013–2014	2011–12	2011		2009	2011
5	Iran (Islamic Republic of)	2015	2015	2017	2015			2014
6	Iraq		2017	2015	^a^			2012
7	Jordan	2017		2007	2010			2007
8	Kuwait			2014	2013			2015
9	Lebanon		2011	2008	2013			2016
10	Libya			2009	^a^	2016		2007
11	Morocco	2003–4			2012			2016
12	Oman		2014	2006	2011			2015
13	Pakistan	2017	2016–17	2014	2013		2014	2016
14	Palestine		2011	2010–11	2011			2010
15	Qatar		2012	2012	2013		2013	2016
16	Saudi Arabia			2005	2013			
17	Somalia		2011		^a^	2016		
18	Sudan	1989–90	2014	2005	2009	2012		2011
19	Syrian Arab Republic			2003	2010			2010
20	Tunisia	1988	2011–12 (2017–18 designing)		2010			2007
21	United Arab Emirates				2015			2015
22	Yemen	2013	2006		2006			2014

^a^The exact year is not confirmed

Therefore, the aim of this study is to identify health indicators that can be effectively obtained from population-based surveys and provide guidance on the surveys needed to generate data for these indicators.

## Methods

### Study design

Previous experiences in the Region especially the experiences of Iran in designing and implementing the health observatory and survey timetable [16, 17] was used as a guide to design this study.

The study was designed as a multistage research process in an exploratory approach to identify the survey-based core health indicators for monitoring the health situation and health system performance in the region, as well as health-related Sustainable Development Goals (SDGs) and their preferred sources. We tried to develop and propose a methodology that can be applicable for other indicators and in other Regions and countries.

#### Data sources

### Primary data sources

The "framework for health information systems and core indicators for monitoring health situation and health system performance" and the SDGs were used as primary data sources.

### Questionnaires and survey websites

Data from questionnaires and websites of eight surveys were collected to identify survey modules and previously conducted surveys in each regional country.

### WHO reports

The WHO 2016 annual report on regional core health indicators and the WHO regional health observatory were used as additional sources of data.

#### Data analysis and synthesis

Survey-based indicators obtained from the primary data sources and survey modules obtained from questionnaires and survey websites were used as sources of data. Each indicator that could be obtained from population-based surveys was identified along with the appropriate module and the corresponding survey (s). Indicators that were not reported in the 2016 WHO report were identified for each country.

#### Dialogue

Expert opinions were obtained and reviewed during a consultative meeting. A timetable for conducting population-based surveys was proposed and finalized based on their recommendations.

The study was conducted in five phases:

1. *Selecting the survey-based indicators:* In order to define the scope of the project, the Regional “framework for health information systems and core indicators for monitoring health situation and health system performance” report [7] and health-related Sustainable Development Goals (SDG) 3 [5] were reviewed by the research team and all their indicators were extracted.
2. A list of 79 indicators containing 68 regional core health indicators and 11 SDG 3 indicators that were not in the regional core indicators list were prepared. Then for each indicator, the preferred source of data was specified. The regional core health indicators and SDG 3 indicators were then categorized into two groups based on their preferred sources of data. 1) indicators that can be obtained from population-based surveys; and 2) indicators that cannot be obtained from population-based surveys, which means that they either can be obtained from administrative data such as death registries or institution-based surveys such as Service Availability and Readiness Assessment (SARA).
3. *Extracting and comparing the survey modules:* In order to identify the surveys that can provide data for the selected indicators and the overlaps between the surveys, relevant modules from main health-related population-based surveys were extracted and compared using the surveys questionnaires as the sources of data. The following eight surveys were assessed: 1) Tunisian Health Examination Survey (HES); 2) Multiple Indicator Cluster Survey (MICS); 3) Demographic and Health Survey (DHS); 4) STEPwise approach to Surveillance (STEPS) survey; 5) Household Expenditure Survey; 6) Global Adult Tobacco Survey (GATS); 7) Global Youth Tobacco Survey (GYTS); and 8) Global School-Based Student Health Survey (GSHS) [18–24]. In addition, we explored the websites for these surveys in order to identify the surveys that were previously conducted in each Regional country and therefore, the countries that have experiences with them (Table 1).
4. *Identifying the sources for the indicators:* Using data gathered in the previous phases, for each indicator that can be obtained from population-based surveys, the appropriate module and the corresponding survey(s) were identified.
5. *Assessing countries status in reporting on core indicators:* By reviewing the WHO 2016 annual report on regional core health indicators and data obtained from the WHO regional health observatory, the missing core indicators in the 2016 report for each country were identified.
6. *Review of results by experts:* The results were presented to and reviewed by the experts during a consultative meeting in Cairo, Egypt, 11–12 December 2017. During the meeting, the findings were presented to the participants and then their opinions were taken in focused group discussions. Based on their opinions a time table for conducting population-based survey was proposed and finalized. The participants were academics with related expertise as well as the members from appropriate bodies in the ministries of health from regional countries. The meeting was moderated by the department of Information, Evidence and Research, WHO Regional Office.

## Results

### Identifying indicators

The review of indicators showed that 44 (65%) out of 68 Regional core health indicators and two (18%) out of 11 SDG 3 indicators not covered in the regional core health indicators list, can be obtained from population-based surveys (Table 2). The indicator "Percentage of individuals who slept under an insecticide threatened bednet the previous night" is only applicable to countries with high risk of local transmission of malaria and is provided by the technical unit in the WHO Regional Office, so it was not included in Table 2. Although data to generate mortality indicators can be obtained from DHS or MICS, it is important to emphasize that the preferred source of data for mortality rates such as neonatal, infant, under-five and maternal mortality is death registry information and surveys are considered as the alternate source [25].

**Table 2:** Survey-based regional core health and SDG 3 indicators by survey modules and the survey(s) that contains the module

Group^a^		Indicator	Survey module	Survey(s)
Demographic and socioeconomic determinants	1	Total fertility rate	Household information	HES/DHS/MICS
	2	Adolescent fertility rate (15–19 years)	Household information	HES/DHS/MICS
	3	Net primary school enrolment	Household education	HES/DHS/MICS
	4	Population below the international poverty line	Household income/expenditure	HES/Household Expenditure Survey
	5	Literacy rate among persons 15–24 years	Household education	HES/DHS/MICS
	6	Access to improved drinking water	Household information	HES/DHS/MICS
	7	Access to improved sanitation facilities	Household information	HES/DHS/MICS
Life expectancy and mortality	8	Neonatal mortality rate	Household information	HES/DHS/MICS
	9	Infant mortality rate	Household information	HES/DHS/MICS
	10	Under-five mortality rate	Household information	HES/DHS/MICS
	11	Maternal mortality ratio	Household information	DHS/MICS
Risk factors	12	Low birth weight among newborns	Children under-5	HES/DHS/MICS
	13	Exclusive breastfeeding rate 0–5 months of age	Children under-5	DHS/MICS
	14	Children under-5 who are stunted	Children under-5 anthropometry	DHS/MICS
	15	Children under-5 who are wasted	Children under-5 anthropometry	DHS/MICS
	16	Children under-5 who are overweight	Children under-5 anthropometry	DHS/MICS
	17	Children under-5 who are obese	Children under-5 anthropometry	DHS/MICS
	18	Overweight (13–18 years)	Children age 13–18 years	GSHS
	19	Obesity (13–18 years)	Children age 13–18 years	GSHS
	20	Overweight (18 + years)	Adult anthropometry	HES/STEPS
	21	Obesity (18 + years)	Adult anthropometry	HES/STEPS
	22	Tobacco use among persons 13–15 years	Children age 13–18 years	GSHS
	23	Tobacco use among persons 15 + years	Adult tobacco	HES/STEPS
	24	Insufficient physical activity (13–18 years)	Children age 13–18 years	GSHS
	25	Insufficient physical activity (18 + years)	Adult physical activity	HES/STEPS
	26	Raised blood glucose among persons 18 + years	Adult laboratory tests	HES/STEPS
	27	Raised blood pressure among persons 18 + years	Adult laboratory tests	HES/STEPS
	28	Anaemia among women of reproductive age	Adult laboratory tests	HES
Morbidity	29	Estimated number of new HIV infections	HIV	Survey in high risk population
Health financing	30	Out-of-pocket expenditure as percent of total health expenditure	Household expenditure	HES/Household Expenditure Survey
	31	Population with catastrophic health expenditure	Household expenditure	HES/Household Expenditure Survey
	32	Population impoverished due to out-of-pocket health expenditure	Household expenditure	HES/Household Expenditure Survey
Health information system	33	Birth registration coverage	Household information	HES/DHS/MICS
	34	Death registration coverage	Household information	HES/DHS/MICS
Service delivery	35	Annual number of outpatient department visits, per capita	Health utilization	HES
Service coverage	36	Demand for family planning satisfied with modern methods	Adult women	HES/DHS/MICS
	37	Antenatal care coverage (1 + ; 4 +)	Adult women	HES
	38	Births attended by skilled health personnel	Adult women fertility-birth history	HES/DHS/MICS
	39	Children under 5 with diarrhoea receiving oral rehydration therapy	Children under-5	DHS/MICS
	40	DTP3/Pentavalent immunization coverage rate among children under 1 year of age	Children under-5 immunization	HES/DHS/MICS
	41	Measles immunization coverage rate (MCV1)	Household expenditure	HES/DHS/MICS
	42	Coverage of service for severe mental health disorders (Denominator)	Mental health disorders	Mental health survey
	43	Percentage of population sleeping under insecticide treated nets	Adult malaria	DHS/MICS
	44	Percentage of key populations at higher risk who have received an HIV test in the past 12 months and know their results	HIV history	HIV survey in high risk population
SDG3	45	Hepatitis B incidence per 100,000 population	Hepatitis B serology	Serology survey for Hepatitis B
	46	Coverage of treatment interventions for substance use disorders (Denominator)	Substance use disorders	Mental health survey

*DHS* Demographic and Health Survey, *GATS* Global Adult Tobacco Survey, *GSHS* Global School-based Student Health Survey, *GYTS* Global Youth Tobacco Survey, *HES* Health Examination Survey, *MICS* Multiple Indicator Cluster Survey, *SDG* Sustainable Development Goals, *STEPS* Noncommunicable Disease Risk Factors Survey

^a^According to the grouping in the "Eastern Mediterranean Region: Framework for health information systems and core indicators for monitoring health situation and health system performance–2016" (7)

### Mapping surveys

As seen in Table 2, these 46 survey-based indicators then were sub-categorized according to the survey modules they can be obtained from and the survey(s) that contains the module(s). The HES could generate data to cover most indicators including all the indicators that can be obtained from STEPS, Household Expenditure Survey and GATS. Thirty (65%) out of 46 indicators can be covered by HES whereas 24 (52%) out of 46 indicators can be covered by DHS/MICS which has 16 overlaps with HES. Six (13%) out of 46 indicators can be covered by STEPS; but all can also be obtained from HES. Four (9%) indicators can be covered by GSHS. Another four (9%) indicators can be covered by Household Expenditure Survey; and all these indicators can also be covered by HES. Two (4%) indicators can be covered by surveys targeting high risk populations for HIV/AIDS. Another two (4%) indicators can be covered by Mental Health Survey although one of them could somehow be covered by HES; and another indicator (Hepatitis B incidence per 100,000 population) requires a serology survey for Hepatitis B.

**Fig. 1:**
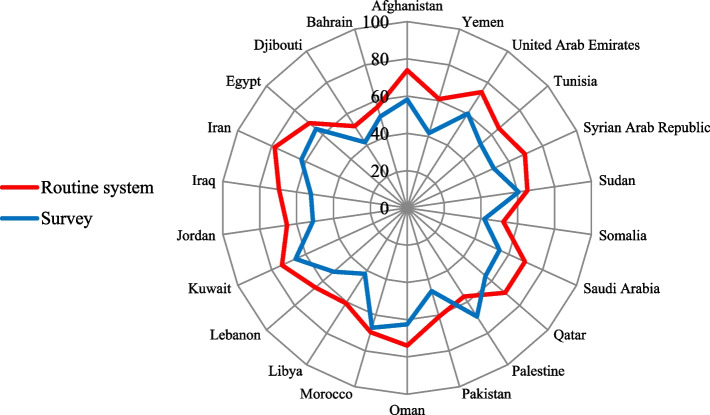
Percentage of regional core health indicators reported by member states for the 2016 report based on the sources of the indicators

Furthermore, the review of the 2016 annual report on regional core health indicators for each country is summarized in Fig. 1. Results show that there are relatively more indicators reported that use data from routine HIS than survey-based indicators. Finally, during the expert consultative meeting, the following key issues were discussed in separate working groups: 1) review and validated the main findings about the last surveys that were conducted in the countries (Table 1), and the indicators and their sources (Table 2), and 2) recommended a list of the population-based surveys for better reporting of core health indicators and SDG3 indicators, as well as the ideal inter-survey period.

During the expert consultative meeting, the following key issues were discussed in separate working groups: 1) review and validated the main findings about the last surveys that was conducted in the countries (Table 1), and the indicators and their sources (Table 2), 2) recommended list of the population-based surveys for better reporting of core health indicators and SDG3 indicators, as well as the ideal inter-survey period.

## Discussion

Our study showed that 44 (65%) out of 68 of the regional core health indicators are obtainable using the data from population-based surveys and the rest need to be gathered by the registration data and routine system. It must be noted that the line between these two groups of indicators is somehow blurry and some indicators can be generated using data from both routine and population-based survey sources. For these indicators, the routine system may be preferred over surveys [26, 27]. However, since many countries lack a robust HIS to gather timely and accurate registration data [28], surveys are usually the default sources of data.

Our findings showed that Health Examination Survey (HES) could cover 65% of the survey-based indicators, and could cover all the indicators that can be obtained from STEPS, Household Expenditure Survey and GATS. Therefore, it is recommended that HES be considered as the main survey in national survey timetables. Further analysis showed that 42 (91%) out of 46 survey-based indicators could be covered by a combination of three surveys (HES, DHS/MICS, and GSHS).

The four indicators that are not covered by these three surveys are as follow:

- 1 & 2. "Estimated number of new HIV infections" and "Percentage of key populations at higher risk (people who inject drugs, sex workers, men who have sex with men) who have received an HIV test in the past 12 months and know their results": In order to obtain these indicators, a survey of high-risk populations is required. Although both indicators could be covered by a single survey.
- 3. "Coverage of services for severe mental health disorders": Although the HES questionnaire contains questions about major depression, the denominator for this indicator requires mental health surveys in order to obtain the prevalence of severe mental health disorders.
- 4. "Hepatitis B incidence per 100,000 population" requires a serology test, which can be added to the HES laboratory test module.

One of the popular surveys in the regional countries is DHS/MICS [29, 30]. Since there are many overlaps between HES and DHS/MICS, conducting both of these surveys in a country is not ideal. One of the most appropriate solutions would be to add relevant modules from DHS/MICS to HES. The following indicators can be generated using data from DHS/MICS as they are not covered by HES:

1. Children under-5 with diarrhea receiving oral rehydration therapy: a question to collect this information can be added to HES individual questionnaire.
2. Exclusive breastfeeding rate 0–5 months of age: relevant questions can be added to the HES individual questionnaire.
3. Maternal mortality ratio: the denominator is already covered by HES, but the question to collect data for the numerator can be added to the questionnaire.
4. Anthropometry in children under 5 (to obtain stunting, wasting, overweighting, and obesity indicators): since adult anthropometry is already part of the HES module, if under-5 anthropometry could be added to the survey, indicators can also be obtained.

These recommendations can be considered when planning to update HES modules.

Although conducting a single omnibus survey such as HES instead of multiple single-purpose surveys has many benefits, such as saving resources and enabling countries to conduct multiple thematic analyses using different variables, there are some issues of concern: 1) Since it takes more time to complete an omnibus survey questionnaire, this might lead to errors and low response rates [31, 32]; 2) Larger surveys require much better planning and logistics before and during the surveys [33]; 3) the donors might not be interested in sponsoring an omnibus survey. To address these challenges, the following solutions are suggested: 1) using a multistage data gathering approach and collecting data over a period of at least two days; 2) WHO could work closely with countries to provide needed technical support to effectively implement an omnibus survey; 3) If the national survey plan or timetable is developed by consultation with development partners and other national stakeholders, then it can be used to the advocate the donors to fund the survey. It must be noted that a well-functioning national HIS is one of the main prerequisites for conducting surveys [34, 35].

Based on the data obtained in the study especially the experts’ opinions, a suggested timetable was proposed for conducting national population-based surveys for the countries in the Region. Three principles were considered when designing the timetable: 1) Since most indicators can be covered by HES, it was selected as the hub of the timetable; 2) According to metadata, most indicators especially those that are generated using data from population-based surveys have to be updated every 3–5 years, therefore, it was recommended that the same survey be conducted every five years; and 3) Considering the difficulties in securing the financial and resources to conduct surveys mentioned during group discussions, only one national population-based survey to be conducted in each year. The finalized timetable is presented in Table 3. The timetable contains both the surveys and the intervals between them. We tried to keep the minimum surveys in the timetable that can generate nearly all required indicators.

**Table 3:**
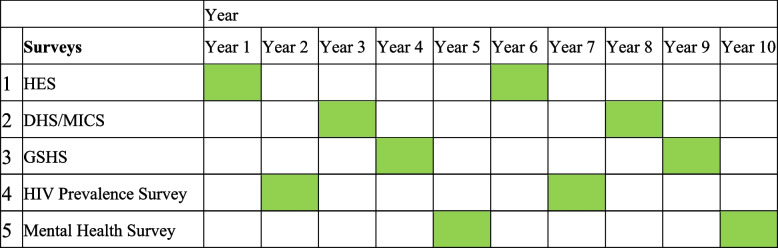
Presentation of the suggested 10-year timetable for conducting national population-based surveys and the intervals between them to obtain the core indicators in priority order

*DHS* Demographic and Health Survey, *GSHS* Global School-based Student Health Survey, *HES* Health Examination Survey, *MICS* Multiple Indicator Cluster Survey, STEPS Noncommunicable Disease Risk Factors Survey

This survey timetable can be implemented in the country in the form of a national charter, which could contain the following: 1) the main steward(s) for conducting each population-based survey in the country; 2) the estimated amount of budget, budget source(s) and how to secure the budget for each survey; 3) the plan for enhancing the secretariat(s) capacity to reliably report on the core health and SDG 3 indicators as well as the public availability survey data. Since many surveys have already been conducted in the countries but the results were not reported to the WHO [9], and 4) as several surveys that are conducted in the Region are not easily accessible or lack clear conditions for access, the charter must also contain a data sharing policy to enhance public access of the data. Further, implementing population-based surveys in the Region at regular intervals can support the validation of some countries' estimates, such as for the global burden of disease when data are calculated and validated across neighboring countries [14, 25, 36].

The high-income countries in the Region that generally have a good electronic HIS, and if needed, they can secure funds to conduct the surveys and implement their own timetable for the surveys. While middle-income countries might need technical and financial support from WHO and other development partners to implement the survey timetable. The low-income countries in the Region mostly lack a robust HIS and may rely on international funds to conduct population-based surveys. This might limit their ability to implement their own timetable, thereby making it crucial to work closely with the WHO in designing and implementing their timetable. However, our findings showed that there were no major differences between high-income and middle and low-income countries in reporting on the core health indicators (Fig. 1).

Also it must be note that as the Region is experiencing some of the worst humanitarian crises [37–40], these crises, along with the political instability and insecurity have affected the coordination, planning and implementation of major data collection activities in the countries.

Most important of all, implementing a plan is far more complicated than designing it. The timetable presented here is just a recommendation, and each country should develop its own tailored timetable. This timetable can be developed and adjusted based on the surveys already conducted in a country in order to provide a good trajectory for the course of surveys and indicators to be generated in the future. The experiences of countries in the Region such as Iran, Sudan, and Qatar that have already developed national survey plans, shows that formal endorsement of the plans by the highest executive authority (i.e. the Minister of Health) can ensure commitment to the national plans [16, 41]. National survey timetables and relevant survey modules should also be reviewed and updated in line with changes or updates in the global, regional or national public health priorities and their monitoring indicators.

## Conclusions

Given that a vast majority (91%) of survey-based indicators can be obtained through the HES, DHS/MICS, and GSHS, these surveys are essential components of national survey plans for reporting health-related indicators in the EMR. Moreover, modifying survey questionnaires can lead to the collection of additional indicators. It is critical to establish an optimal schedule for conducting population-based surveys and to use it as a framework for national planning.

## Limitations

This study mainly focused on population-based surveys that can generate most indicators. However, it must be emphasized that several other factors must be considered when designing and implementing a national survey timetable, such as national development priorities, technical expertise and available resources.

## Data Availability

The summary report on the expert consultative meeting held in Cairo, Egypt, 11–12 December 2017 is available from: https://apps.who.int/iris/handle/10665/260371. The datasets on regional core health indicators reported by countries (Fig. 1) are available from the WHO Regional Health Observatory (https://rho.emro.who.int/). All analytical data and related methods are available from the corresponding author upon request.

## Abbreviations

DHS: Demographic and Health Survey
EMR: Eastern Mediterranean Region
GATS: Global Adult Tobacco Survey
GSHS: Global School-based Student Health Survey
GYTS: Global Youth Tobacco Survey
HES: Health Examination Survey
HIS: Health Information System
MICS: Multiple Indicator Cluster Survey
SARA: Service Availability and Readiness Assessment
SDG: Sustainable Development Goal
STEPS: STEPwise approach to surveillance
UHC: Universal Health Coverage
WHO: World Health Organization
